# A State Medical Service

**Published:** 1918-11-23

**Authors:** 


### THE EDITOR'S LETTER-BOX.

[Correspondence on all subjects is invited, but we cannot in any way be responsible for the opinions expressed by our correspondents, who must give their name and address as a guarantee of good faith, but not necessarily for publication. Correspondents are reminded that brevity of style and conciseness of statement greatly facilitate early insertion.]

### A State Medical Service.

*To the Editor of* The Hospital.

Sir,—I have read Colonel Maurice's article on a State Medical Service with much interest. I am a theoretical believer in a State Medical Service, but I see grave difficulties in the way of its effectual establishment. Possibly these may be overcome. In my experience, which is limited, the only really well-run State Service in the country during our time has been the Navy. Perhaps the Post Office or the Inland Revenue might come in as a good second. The bogey is always family influence. How is it to be guaranteed that the efficient rather than the influential man will get the job? I am no backer of German systems, but it is no harm to observe that before the war German military officers were on the whole more efficient than men of the same rank in the British Army. Our men have been "ragged" for being keen.

If it could be guaranteed that efficiency pure and undiluted would be rewarded, and that the sick man or woman could be sure of the best available treatment, a State Medical Service would be vastly superior to that which obtains at present. Take my own personal experience. My present place of abode I should not like to leave because of my doctor, who is extremely clever and extremely painstaking. Candidly, I do not know where I should find his equal. I have known him to attend a poor patient at noon, to go home to see his own daughter dying, and to come back again at night. We all know what Government officials are. I, at least, know. I am myself a Government official! What guarantee shall we have that official doctors and official nurses will do their duty to the uttermost limit of their ability?—and what guarantee that when vacancies arise Lord Tom Noddy, who is the doctor's uncle, or the local County Council Chairman, who is his father, will not pull all the strings on earth and under the earth in order to secure the advancement of somebody who has no other claim to preferment?

If perfect efficiency can be ensured I am behind Colonel Maurice to the last button. But, candidly, I am in fear and trembling of two things. One is string-pulling, and the other is my own *alter ego*.

Red Tape.

